# Erratum for Deines et al., “Microbial Species Coexistence Depends on the Host Environment”

**DOI:** 10.1128/mBio.03339-20

**Published:** 2021-01-26

**Authors:** Peter Deines, Katrin Hammerschmidt, Thomas C. G. Bosch

**Affiliations:** a Zoological Institute, Christian Albrechts University Kiel, Kiel, Germany; b Institute of General Microbiology, Christian Albrechts University Kiel, Kiel, Germany

## ERRATUM

Volume 11, no. 4, e00807-20, 2020, https://doi.org/10.1128/mBio.00807-20. There was missing information regarding the affiliation of one author. It should appear as shown above.

